# Supplementary material for: networkGWAS: a network-based approach to discover genetic associations
Source: Bioinformatics. 2023 Jun 7;39(6):btad370. doi: 10.1093/bioinformatics/btad370 (PMC10281858; doi:10.1093/bioinformatics/btad370)
Supplement: btad370_Supplementary_Data [file btad370_supplementary_data.zip › Supplementary.pdf]

Supplementary Material

S.1 Covariates as fixed effects

Equation (2) introduces the LMM model that `networkGWAS` relies on, and, in particular, the matrix of the fixed effects X_f. The latter comprises the vector of 1s corresponding to the intercept, but it could also include other additional covariates when needed. In the context of GWAS, these covariates are factors that are expected to have an impact on the studied phenotype and, potentially causing spurious associations. This could result in not calibrated p-values. For example, when studying height or certain diseases in *H. sapiens*, it might be reasonable to include age and sex as covariates since they might affect these phenotypes. Another aspect that could influence the phenotype and cause miscalibration in the distribution of the p-values is the so-called population structure, which we already introduced in the main manuscript. To mitigate the effect of population structure, one could include as covariates in X_f the principal components calculated as in Price *et al.* [29].

S.2 Computational cost

We base our method on the FaST-LMM-Set procedure [18], and in particular, we use the one-variance component model. A single test on a neighborhood using this procedure presents a time complexity of $O(nn_\mathrm{s}^2)$ in two cases, namely (i) when K_s is the linear kernel and the number of SNPs in the test set n_s is less than the number of individuals n, and (ii) when K_s is the polynomial kernel and $n_\mathrm{s}(n_\mathrm{s}-1)/2 < n$, because the trivial factorization in the reproducing kernel Hilbert space of dimension $n_\mathrm{s}(n_\mathrm{s}-1)/2$ has to be obtained. Otherwise, each test costs $\mathcal{O}(n^3)$. Hence, in the best-case scenarios, testing n_g neighborhoods has a runtime of $\mathcal{O}(n_\mathrm{g}nn_\mathrm{s}^2)$, where n_g is the number of genes considered in the analysis. Before applying FaST-LMM-Set, however, `networkGWAS` requires the aggregation of gene neighborhoods, which scales linearly in the number of edges of the PPI employed. As biological networks can be assumed to be only sparsely connected, this leads to a runtime of order $\mathcal{O}(n_\mathrm{g})$. Secondly, in order to obtain p-values from the test statistics, we have to repeat both the neighborhood aggregation and the n_g set tests for each of the n_p permutations. This results in a computational cost of $\mathcal{O}\left(n_\mathrm{p}n_\mathrm{g}nn_\mathrm{s}^2\right)$ if $n_\mathrm{s} < n$ for the linear kernel or $n_\mathrm{s}(n_\mathrm{s}-1)/2 < n$ for the quadratic kernel. Otherwise, the cost amounts to $\mathcal{O}\left(n_\mathrm{p}n_\mathrm{g}n^3\right)$. Therefore, while the cubic scaling is not favorable for large cohort sizes, asymptotically, run time scales linearly in n. For the *S. cerevisiae* data, the median number n_s of SNPs per neighborhood is 215. Hence, for the majority of *S. cerevisiae* PPI neighborhoods tested, the cubic-to-linear speed up sets in for cohort sizes as small as $n = 971$ for linear `networkGWAS`, while for non-linear `networkGWAS` that speed-up would be achieved for cohorts of $n \gtrsim 471,000$ samples. Note that considering the computational time to perform the permutations themselves, i.e. rotating the SNPs and permuting the network, would add $O(n_\mathrm{p})$ and $O(n_\mathrm{p}n_\mathrm{g})$–which becomes $O(n_\mathrm{p}n_\mathrm{g}^2)$ in case of non-sparse network–to the total time complexity respectively, terms that are dominated by the above derived time complexity, regardless of the case.

picture(0,0)(-35,0)(1,0)30 (0,35)(0,-1)30 picture picture(0,0)(35,0)(-1,0)30 (0,35)(0,-1)30 picture

S.3 Model choices: motivation

The log-likelihood of the model underlying the original FaST-LMM-Set [22, 23] method writes:

$$LL(\beta, \sigma_e^2, \tau) = \log \mathcal{N}(\vec{y}|X\vec{\beta}; \sigma_e^2 I + \sigma_g^2[(1-\tau)K_c + \tau K_s])\,, \tag{S.1}$$

and features two random effects: one to capture confounders (K_c) and another to account for similarity among the SNPs of the set to be tested (K_s). The covariance matrices are defined as $K_c = \frac{1}{n_c}V_c V_c^T$ and $K_s = \frac{1}{n_s}V_s V_s^T$, where V_c contains the n_c SNPs from which relatedness is estimated and V_s contains the n_s SNPs to test. As introduced in [19], the parameter $\tau \in [0, 1]$ serves to distinguish the null model (i.e., $\tau = 0$) from alternative models (i.e., $0 \leq \tau \leq 1$), and is estimated from the GWAS data set by means of restricted maximum likelihood. Once the value of equation (S.1) has been calculated for the null and the alternative hypotheses, it is possible to compute the likelihood-ratio test statistics, i.e., $t = -2(LL(\tau = 0) - LL(0 \leq \tau \leq 1))$. Now, the original FaST-LMM-Set follows the spirit of Wilks' theorem [41] and results by Greven *et al.* [13] and employs a mixture of χ^2 distributions,

$$p_0(x) = a\chi_0(x) + b\chi_d(x) \tag{S.2}$$

to serve as parametric distribution of the test statistic under the null hypothesis. The parameters are determined by fitting $p_0(x)$ to the 10% most significant tail of the null distribution of test statistics obtained by permuting individuals for only the SNPs in the set of interest, that is, permuting the rows of V_s.
We chose to implement an alternative permutation strategy for the following reasons. As already mentioned above, K_c is the kernel matrix that captures confounders, and represents the genetic similarity matrix (GSM), which measures and corrects for population structure in the form of a realized relationship matrix (RRM) [12, 15]. K_c is calculated as $\frac{1}{n_c}V_c V_c^T$; the standard, computationally efficient way to choose the SNPs to include in V_c is the so-called "leave-one-out-chromosome" strategy [21], where the sets of SNPs on each chromosome are tested separately and K_c is constructed using the SNPs located on the other chromosomes. Such a strategy is not computationally reasonable when testing neighborhoods, as genes interacting through a biological network can be, and frequently are, located on different chromosomes. Therefore, when excluding the GSM K_c for such reasons from our model, employing FaST-LMM-Set permutation scheme, i.e., permuting the individual of K_s, would result in not preserving the population structure signal when generating the null distribution [16]. Hence, we implemented the permutation scheme described in Section 2.3, which allowed us to preserve population structure when constructing the null distribution when testing neighborhoods.
Furthermore, while fitting a parametric null distribution saves computation time by decreasing the number of permutations needed, we found the resulting estimate of the distribution $p_0(x)$ of test statistics under the null hypothesis did not reflect the true distribution in our case. Similar limitations of the above parametric approach in LMMs have been discussed in [27], and hence we adhere to a non-parametric distribution for `networkGWAS` instead, enabling us to obtain well calibrated p-values across scenarios and simulations, as exemplified in Fig. S.3. More specifically, `networkGWAS` allows us to obtain a genomic inflation factor (defined as the ratio of the median observed to median theoretical test statistic) $\lambda_{GC} \in [0.8, 1.2]$ for 98% of the scenarios analysed in the *A. thaliana* semi-simulation, in 92% of the natural *A. thaliana* phenotypes, in 100% of the *S. cerevisiae* studied phenotypes, and in 67% of the Estonian BioBank traits. For some of the simulated phenotypes in the *H. sapiens* use case, the portion of high p-values present deflation, leading to $\lambda_{GC} \notin [0.8, 1.2]$, while the rest of the distribution of the p-values, including the low-p-values range, look calibrated. Note that we obtained these results without any hyperparameter selection procedure. In fact, our permutation technique requires only one parameter to set, the percentage of edges to permute, which we set to 50%. In contrast, the original FaST-LMM-Set requires an optimization step aimed at selecting the SNPs modeling the relatedness in the dataset, i.e., the SNPs to include in V_c. In fact, to yield the computational savings that reside at the core of the FaST-LMM procedure [21], the sum of the dimensions of the kernel matrices K_c and K_s must be kept well below the number n of samples studied. To this end, the GSM K_c is constructed from a limited number of SNPs chosen on the basis of their p-values in an uncorrected linear regression with respect to the phenotype of interest, following the leave-one-out-chromosome approach. Lastly, to identify the precise number of SNPs to be included in the GSM, the latter is constructed with an increasing number of SNPs, starting with those associated with the lowest p-values, until the first minimum in the genomic inflation factor λ_{GC} is met [21].

picture(0,0)(-35,0)(1,0)30 (0,-35)(0,1)30 picture picture(0,0)(35,0)(-1,0)30 (0,-35)(0,1)30 picture

"output" — 2023/4/7 — page S.2 — #11

S.4 Number of permutations in `networkGWAS`

As detailed in Section 2.3, we obtain our null distribution T_0 by pooling the statistics from each neighborhood from each permutation. Hence, if $n_{\rm g}$ is the number of neighborhoods and $n_{\rm p}$ is the number of permutations, the non-parametric null distribution is composed of $n_{\rm g} \times n_{\rm p}$ tests statistics, decreasing the total number of permutation required. A question now arises, namely "how to define a proper number of permutations $n_{\rm p}$?". There are two criteria that allow one to answer this question. First, one could check the distribution of the null test statistics obtained after each permutation and find after which permutation this distribution stabilizes. Second, our method foresees this hierarchical procedure based on B-H as a means to correct for multiple testing. Hence, the minimum obtainable p-value, which depends on the number of permutations, should be "small enough" to allow for discovering significance (if any). In practise, we found that a number of permutations among 100 and 300 is a good compromise to reduce the required computation and have a valuable estimate of the p-values.

S.5 Hierarchical testing procedure

Correction for multiple testing is necessary when dealing with a multitude of hypotheses, such as in GWAS, where millions of genetic variants are studied and tested at once. In such scenarios, the false discovery rate (FDR) is often used as a measure of global error. FDR is defined as the expected proportion of findings, i.e., rejected null hypotheses, for which the null hypothesis is actually true. As defined in Section 2.3, the null hypothesis H_j for `networkGWAS` signifies that the j-th neighborhood $\mathcal{N}_k(v_j)$ does not affect the phenotype. When studying P related phenotypes (for example phenotypes obtained from the same study), we can rewrite the null hypothesis as H_{jt}, and it represents the fact that the j-th neighborhood does not affect the t-th phenotype. This results in having a collection of null hypotheses $\{H_{jt}, j = 1, \ldots, M; t = 1, \ldots, P\}$. Having all these hypotheses from different neighborhoods and phenotypes, one possibility would be to control the global FDR, e.g., applying the Benjamini-Hochberg (B-H) procedure [3] on the pooling of the hypotheses. However, this approach has been shown to present a suboptimal behaviour, that is, for phenotypes presenting no or low association signal, the FDR would not be controlled at the defined level q, while for phenotypes showing medium or high association signal, this procedure would cause a decrease in the power [9]. Peterson *et al.* [26] define a rigorous procedure aimed at correcting the aforementioned situation, which will be detailed in the following. Consider again the collection of null hypotheses $\{H_{jt}, j = 1, \ldots, M; t = 1, \ldots, P\}$. Among them, we can identify subgroups of hypotheses, based on what we are interested in studying. In our case, one group corresponds to all the hypotheses that relate to one phenotype, and we define each group as $\mathcal{P}_t = \{H_{jt}, j = 1, \ldots, M\}$. Note that when introducing the concept of group/subgroups in the null hypotheses collection, Peterson *et al.* [26] refer to them as "families"; here we use these terms interchangeably. From these families, we can obtain a global null hypothesis, which in our case writes $H_{\bullet t} = \cap_{t=1}^{M} H_{jt}$ and means that no neighborhood affects the t-th phenotype. Having defined the notation, we can introduce the procedure, which is comprised of three steps, namely:

1. Employing Simes's method [34] to calculate a p-value per each global hypothesis, i.e., $H_{\bullet t}$. Precisely, these P p-values ($p_{\bullet t}$) are calculated as:
$$p_{\bullet t} = \min_{j=1,\ldots,M} M p_t(j)\,, \tag{S.3}$$
where $p_t(j)$ is the p-value obtained for the neighborhood j for the phenotype t.
2. Applying the B-H procedure, with FDR target set to q_1, on the p-values obtained according to point 1. The set of phenotypes for which the null hypothesis $H_{\bullet t}$ has been rejected is defined as $\mathcal{S}$.
3. For the phenotypes in $\mathcal{S}$, applying B-H on their respective subgroup of hypotheses, $\mathcal{P}_t$, with FDR target set to $q_1 * |\mathcal{S}|/P$. This step returns the statistically associated neighborhoods for the phenotypes in $\mathcal{S}$, i.e., those phenotypes that reject the null hypothesis of no neighborhood affecting them.

When Simes's p-values are valid [31], the described procedure guarantees the control of the FDR for the discovery of the phenotypes (vFDR in [26]), and when B-H is applied on the set of global hypotheses, then it controls FDR at level q_1, allowing to identify $\mathcal{S}$ while indeed controlling the FDR. Then, it controls the average FDR on the families in $\mathcal{S}$ (sFDR in [26]), at level q_2, by applying B-H as described in point 2 above.

S.6 Data

S.6.1 Common variants semi-simulations

We use the genotype dataset available on the manually curated and standardized GWAS catalog for *A. thaliana*, i.e., AraGWAS [39], and in particular, we utilize the fully-imputed dataset, which comprises 2,029 samples with 10,709,466 SNPs each. We filter the SNPs for at least 5% minor-allele frequency, leaving us with 1,763,004 SNPs. Out of those, 37,458 can be mapped to the 1,327 genes included in the PPI network, obtained from The Arabidopsis Information Resource (TAIR) database [18] using the strict (i.e., without windows of base pairs) positional mapping of the genes downloaded from TAIR. We chose the TAIR network for our semi-simulated setting since its smaller size allowed us to run a large number of fast experiments.

S.6.2 Rare variants simulations

With the aim of simulating rare variants with realistic LD and MAF distributions, we use the sim1000G package [8], which requires the variant call format (VCF) file of the genomic region of interest as the unique input. We choose the VCF file for chromosome 10 from Phase III 1000 genomes sequencing data [2], and we use PLINK [30, 33] to extract the variant calls for the European population. We employ bcftools [7] to further manipulate the VCF file, namely to filter out the variants that are not mapped – according to the MyGene database [42, 43] – onto the 398 interacting genes through the human STRING PPI network [35]. This is always restricted to chromosome 10. Note that working on a subset of the *H. sapiens* genes does not compromise the results since the genotype-phenotype association signal is simulated. Having obtained the desired VCF file, we therefore use sim1000G to simulate 10,000 rare variants, i.e. MAF $\leq$ 0.1, from 500 unrelated subjects. The generation of the synthetic phenotypes follows the same steps and parameter choices as detailed in Section S.7, apart from n_{cg}, which is set to 15, 8 or 2 to have the same causal/non-causal genes ratio as for the *A. thaliana* use case. Lastly, the simulation settings S0 to S10 are chosen following the common-variant semi-simulations listed in Table S.2. Comparison partners and performance evaluation follow what reported in Section 3.2.

S.6.3 *A. thaliana*

As reported in the main text, when studying natural phenotypes from *A. thaliana*, we again use the genotype data from the AraGWAS Catalog [39], but rely on the larger STRING database for the PPI network [35], considering the high confidence PPIs. In fact, the interactions in the STRING PPI network are annotated with one or more "scores", which are indicators of confidence, i.e. how likely STRING judges an interaction to be true given the available evidence. These scores rank from 0 to 1, with 1 being the highest possible confidence. For our analysis we only consider high-confidence interactions with a score larger than or equal to 0.7. The phenotypes are selected from the AraPheno [32] database, a central repository of population-scale phenotypes for *A. thaliana* inbred lines, based on the criteria that they are quasi-continuous (such as stem length, and flower diameter) and be known for at least 500 out of the 2,029 *A. thaliana* samples that we use the genotypes of. This leads to the selection of 13 phenotypes, summarized in Table S.3. Note that to date there are no significantly associated genetic variants reported on the AraGWAS Catalogue for the analyzed phenotypes. For each phenotype, we collect the fully-imputed genotypes provided by AraGWAS for which the phenotype of interest has been determined. We then filter for at least 5% minor-allele frequency among those samples which, depending on the phenotype, leaves us with 1,837,440 $\pm$ 27,477 remaining SNPs. Out of those, 968,620 $\pm$ 16,200 could be positionally mapped [18] to the 31,568 $\pm$ 18 *A. thaliana* genes that present at least one SNP mapped to it. Note that isolated genes according to the STRING PPI network are considered in the analysis.

S.6.4 *S. cerevisiae*

When studying *S. cerevisiae*, we obtain both the GWAS dataset and the phenotypes from the study by Peter *et al.* [25]. The GWAS dataset comprises 1,011 *S. cerevisiae* isolates of 83,794 genetic variants (82,869 SNPs and 925 copy-number variants, MAF $\geq$ 0.05) each. For 971 of the 1,011 strains, the growth ratio between 35 stress conditions and the standard growing condition for *S. cerevisiae* has been recorded. Among these 35 phenotypes, we consider the 23 that present a Gaussian distribution of the values, listed in Table S.4. The *S. cerevisiae* PPI network has been downloaded from the STRING database [35], and we again only consider high confidence interactions, i.e., interactions with confidence score greater or equal than 0.7, in order to limit the bias due to the noise/uncertainty of the PPIs. After performing the SNPs-genes strict positional mapping [6], 59,787 SNPs and 5,737 genes remain.

S.6.5 *H. sapiens*

The Estonian BioBank GWAS dataset is comprised of 38,226,084 genetic variants (assembly GRCh37) across 202,910 samples. After filtering at MAF threshold 0.05, we remain with 5,968,104 SNPs. We then positionally map the SNPs to the protein coding genes, whose list has been retrieved from the GENCODE database [11]. The gene annotations utilized as mapping between the SNPs and the genes has also been obtained from the GENCODE database [11]. This mapping operation leaves us with 2,565,733 SNPs. Finally, to reduce collinearity, SNPs are pruned based on linkage disequilibrium to preserve 90% of their total variance. 674,548 is the final number of SNPs considered in our analysis, and 18,048 is the amount of genes presenting at least one SNP mapped on it. Whereas for the PPI network, we select high confidence PPIs (i.e., PPIs presenting confidence score $\geq$ 0.7) for the 18,048 selected protein coding genes from the STRING database [35].
As already introduced in the main text, we study three phenotypes: (i) type II diabetes diagnosis, which presents 199,466 samples; (ii) height, whose cardinality is 148,144; and (iii) and BMI, with 136,772 samples. In addition to the above annotations, each sample presents additional information, such as sex and age at time of diagnosis.

S.7 Phenotypes simulation

As mentioned in the main text, to simulate the phenotypes, we firstly define the following parameters:

- the number of genes n_{cg} that carry causal SNPs, henceforth called causal genes. This parameter serves to distinguish between phenotypes governed by a high number of genes from phenotypes influenced by a few genes;
- the ratio of causal SNPs on a causal gene, r_{c}. This parameter governs the percentage of the subset of SNPs on a gene that contributes to the association signal;
- the mean ratio of causal neighbors (RCN) of a causal gene. This parameter allows us to define situations where either none or a small part or a large part of the neighbors of a gene carries association signal;
- the signal-to-noise-ratio (SNR), which reflects on the heritability of the phenotype; and
- the mixing ratio of linear-to-nonlinear (RLN) signal, to differentiate between situations where the association signal is purely linear, or where the association signal is generated from the interaction between the SNPs, or a mixture between these two extremes.

All the above defined parameters are systematically varied in our experiments. Thus, we investigate the robustness of our method's and comparison partners' performance as we depart from the most amenable scenario (S0) of a purely linear signal, spread across a single or very few causal subgraph(s) composed of a high number of causal genes, with a high ratio of causal neighbors, a realistic signal-to-noise ratio, and a high ratio of causal SNPs on the causal genes. Table S.2 summarizes the scenarios simulated, starting from our anchor scenario S0 and deviating from its conditions by:

- Varying the SNR while keeping the RCN, RLN, r_{c}, and n_{cg} constant,
- Varying the RLN while keeping the SNR, RCN, r_{c}, and n_{cg} constant,
- Varying the RCN while keeping the SNR, RLN, r_{c}, and n_{cg} constant,
- Varying the r_{c} while keeping the SNR, RCN, RLN, and n_{cg} constant,
- Varying the n_{cg} while keeping the SNR, RCN, RLN, and r_{c} constant.

We thereby study the isolated effects of moving towards more challenging/different RCN, RLN, SNR, r_{c}, and n_{cg} respectively.
In order to realize the predefined scenarios, we follow the procedure below for the simulation of the artificial phenotype:

1. Choice of causal genes:

   a. For RCN equal to 0./0.4/0.8, randomly select n_{cg}/5/1 node(s) in the network and define them as causal.
   b. Randomly include 0%/40%/100% of the k-hop neighbors of the starting node(s), then 0%/40%/100% of the k-hop neighbors of the newly included nodes and so on, until the pre-defined number n_{cg} of causal genes is reached.
   c. Should the starting node(s) belong to a disconnected subgraph(s) smaller than as to allow for the inclusion of n_{cg} causal genes, repeat (1)-(3) until n_{cg} causal genes have been defined.
   d. Compute RCN, and if RCN is not in $[0.]$ / $[0.4, 0.5]$ / $[0.8, 1.0]$, disregard and repeat (1)-(4).

2. Choice of causal SNPs and causal interactions:

   a. Randomly select a ratio of r_{c} of the SNPs of each causal gene as carrying the signal.
   b. For each causal subgraph, define one of the nodes with highest degree as the center of the subgraph. For each causal SNP belonging to a non-center node of the same causal subgraph, randomly select a causal SNP on the center node and define the interaction between the two SNPs as causal. Ensure that each center-node causal SNP has at least one interaction partner.

3. Computation of the artificial phenotype:

   a. Simulate the phenotype as
   $$\vec{y} = cX \cdot \vec{\beta} + (1-c)X^{(2)} \cdot \vec{\beta}^{(2)} + \vec{\epsilon} \tag{S.4}$$
   where X is the n by p matrix of all SNPs across the genes considered in the analysis (i.e., 37,458 in the semi-simulated case and 10,000 in the simulated case), $\vec{\beta}$ represents the fixed effect of these SNPs, $\vec{\epsilon}$ models the noise, $X^{(2)}$ is the n by $p(p-1)/2$ second-order design matrix of all SNP interactions, $\vec{\beta}^{(2)}$ comprises the fixed effects of all SNP interactions, and the coefficient c serves to tune between the ratio of linear to non-linear signal. $\vec{\epsilon}$ is drawn from a multivariate normal distribution $\mathcal{N}(\vec{0}, I)$.
   b. Set β_i to a fixed and positive value b if the ith SNP is a causal one, or to zero otherwise. Choose b such that for $c = 1$, the SNR equals the desired value.
   c. Set $\beta_i^{(2)}$ to a fixed and positive value $b^{(2)}$ if the ith SNP-interaction is a causal one, or to zero otherwise. Choose $b^{(2)}$ such that for $c = 0$ the SNR equals the desired value.
   d. Choose c such that the desired RLN is realized.

Tuning the signal-to-noise ratio (SNR) in our simulation allows us to mimic a range of heritability values h by means of the relation $\text{SNR} = h/(1-h)$. With low but realistic heritability values ranging from $h = 0.1$ to $h = 0.4$, SNRs to be investigated stretch from $\text{SNR} = 0.1$ to $\text{SNR} = 0.67$, which includes all the values investigated by us. Lastly, note that we create 5 random realizations of each scenario S0-S10, in order to estimate the variance in performance.

S.8 Results on the rare variants simulations

Having assessed `networkGWAS`'s ability of successfully detecting known associations on the common variants semi-simulated use case, we decided to test `networkGWAS` on yet a different scenario, namely on synthetic rare variants and phenotypes. The synthetic genotype, simulated phenotypes and PPI network we utilize in these experiments are described in Section S.6.2.
The results obtained for the rare-variant simulations are summarized in Figure S.2 and are very similar in nature to the results obtained on common variants. As can be seen in the top left panel, in our scenario S0 of a purely linear signal, spread across a single or very few causal subgraph(s) with a high ratio of causal neighbors, and high signal-to-noise ratio, both linear and non-linear `networkGWAS` outperform all the comparison partners by achieving an AUPRC of 69.9%$\pm$17.9% and 65.6%$\pm$18.4%, respectively. As demonstrated in panel (b) and (c) of Figure S.2, this dominance is maintained while varying SNR and linear-to-nonlinear signal mixtures, and only vanishes for a fully nonlinear phenotype. Note, however, that in the rare variants case, `networkGWAS` is less robust to both increasing SNR and increasing nonlinearity of the signal than for the common variants. Furthermore, for RVs, the non-linear SNP-set kernel does not perform better than the linear one in any of the scenarios studied. As in the common-variant simulations, in the rare-variant case, too, `networkGWAS` cannot outperform its comparison partners in the case of isolated causal-genes, while `networkGWAS` does outperform comparison partners as soon as the PPI network holds phenotype-relevant information. This is shown in panel (d) of Figure S.2. Similar considerations to the *A. thaliana* use case apply to the variation of the ratio of causal SNPs on the causal genes and of the number of causal genes; results shown in the panels (e) and (f) of Figure S.2.

S.9 Application to *H. sapiens*: supplementary

As anticipated in the main text, we employ `networkGWAS` and its comparison partners on human genetics data from the Estonian BioBank [19], a volunteer-based cohort of the Estonian adult population, with more than 200,000 participants of age 18 and over. Here, we report more comprehensive results on the three analyzed phenotypes.

Type II diabetes The first phenotype we analyze consists in the presence or absence of a type II diabetes diagnosis. Specifically, this is a binary phenotype where 1 indicates the presence of type II diabetes diagnosis, either at recruitment (prevalent T2D) or during follow-up (incident T2D, data obtained via Electronic Health Record linkage), whilst 0 represent individuals without any diabetes-related diagnosis, since individuals with type I diabetes have been removed from the analysis. There is a total of 14,701 type II diabetes cases and 184,765 controls. Theoretically, it would be more appropriate to utilize a generalized linear model, for example logistic regression, instead of LMM for analyzing such a phenotype. In this setting, however, exact inference for logistic models is generally intractable. This requires the use of approximations, for instance the Laplace approximation to the quasi likelihood [4], which is commonly used in practise. Lippert *et al.* [22] compared the use of this approximation to the use of a linear model, less appropriate but exact, and observed very little difference, if any, between them. Hence, following [22], we use our LMM-based approach on such phenotype.
As seen in the main text, the analysis of this phenotype allows to identify 9 significantly associated neighborhoods, which compose a subgraph on the PPI network, with 144 nodes, i.e., genes. According to the enrichment analysis performed using the WEB-based GEne SeT AnaLysis Toolkit (WEBGESTALT) [40], we found out that 42 genes are already known to be involved in type II diabetes-related processes (see the Supplementary files for the detailed results). WEBGESTALT directly allows to query diseases databases and, specifically, we queried:

- DisGeNET [28], which reports gene-disease associations from curated repositories, GWAS catalogues, animal models and literature;
- Gene List Automatically Derived For You (GLAD4U) [17], that contains gene-disease links extracted from PubMed literature; and
- Online Mendelian Inheritance in Man (OMIM) [14], the database of associations between genes and Mendelian phenotypes.

The comparison with the baselines reports that:

- traditional GWAS identifies 1 among the 102 candidates, and 12 among the known 42 genes;
- NAGA is capable to detect 4 out of the 102 candidates, and 17 out of the 42 known genes;
- dmGWAS finds 5 of the 102 candidates, while it is capable of surfacing 14 of the 42 known genes;
- finally, FaST-LMM-Set (genes), instead, does not report statistically significant results.

This shows that the remaining 102 candidates genes identified by `networkGWAS` are only limitedly, i.e., 7.84%, surfaced by the comparison partners, whilst a good proportion (47.62%) of the already known genes are partially detected by the baselines as well.
A preliminar analysis of these candidates genes reports that many of them are involved in the Wnt signaling, which is known to be related to diabetes type II [5]. Additionally, we, for example, find out that among the candidate genes uniquely identified by `networkGWAS`, that *ENSG00000165819* and *ENSG00000165819* are involved in the endothelial to hematopoietic transition, process that seems to be related to diabetic retinopathy [20]. Furthermore, *ENSG00000177606*, *ENSG00000087095*, *ENSG00000170345*, *ENSG00000020633*, and *ENSG00000196591* are involved in the response to transforming growth factor beta, processes related to diabetic nephropathy [1]. These examples suggests that further investigating these candidates genes might uncover new insights underlying type II diabetes cellular processes.

Height & BMI On these phenotype, `networkGWAS` reports no statistically significant neighborhoods. The traditional GWAS analysis, however, finds statistical associations located on multiple genes, for both phenotypes. These results show that it might not always be beneficial to incorporate the biological network information: analyzing the phenotypes under different complementary perspectives could improve the understanding of the obtained results.

S.10 Statistically associated SNPs, genes and neighborhoods

S.10.1 Univariate GWAS hits on *A. thaliana* phenotype 704

The genes where the SNPs identified by traditional univariate GWAS analysis are located are *AT4G00630, AT4G00730, AT4G00740, AT4G00752, AT4G01010, AT5G45750,* and *AT5G45760.* We compare these findings with the results available on the AraGWAS Catalog [39] for phenotype 704, namely the number of rosette leaves. To ensure a fair comparison, we filter the published associations using the same MAF threshold we employed, we consider only genic SNPs, and we use FDR for multiple testing correction. This leads to have no significant associations on the AraGWAS Catalogue for phenotype 704. To biologically investigate these findings, we use the gene ontology (GO) Term Enrichment for Plants [36] provided by TAIR, which relies on the PANTHER Classification System [24, 38]. We use the PANTHER Over-representation Test with the same settings as done for *S. cerevisiae*. This analysis does not yield to any significantly enriched process. However, the TAIR database reports that *AT4G00630*, *AT4G00740*, and *AT5G45750* are expressed in the rosette leaves, therefore representing biologically plausible results.

S.10.2 `networkGWAS` (K_{lin}) on *S. cerevisiae* phenotype YPGALACTOSE

The two identified neighborhoods, namely *YGR252W*'s and *YNL031C*'s, present 74 overlapping genes, and their union is composed of 265 genes in total, which are:
YAL013W, YAL019W, YAR003W, YAR007C, YBL002W, YBL003C, YBL008W, YBL023C, YBL052C, YBL088C, YBL103C, YBR034C, YBR081C, YBR103W, YBR109C, YBR112C, YBR114W, YBR133C, YBR136W, YBR160W, YBR175W, YBR195C, YBR198C, YBR202W, YBR215W, YBR245C, YBR274W, YBR289W, YCL010C, YCL061C, YCR012W, YCR033W, YCR065W, YCR082W, YCR084C, YDL003W, YDL017W, YDL042C, YDL074C, YDL139C, YDL140C, YDR013W, YDR034C, YDR052C, YDR083W, YDR096W, YDR145W, YDR167W, YDR176W, YDR181C, YDR190C, YDR191W, YDR216W, YDR217C, YDR224C, YDR227W, YDR303C, YDR334W, YDR359C, YDR392W, YDR440W, YDR448W, YDR469W, YDR477W, YDR489W, YEL009C, YEL018W, YEL021W, YEL032W, YEL056W, YER040W, YER051W, YER052C, YER095W, YER110C, YER148W, YER164W, YER169W, YFL021W, YFL024C, YFL037W, YFL039C, YFR037C, YGL008C, YGL043W, YGL058W, YGL060W, YGL066W, YGL073W, YGL112C, YGL116W, YGL150C, YGL163C, YGL194C, YGL201C, YGL207W, YGL237C, YGL244W, YGL252C, YGR002C, YGR056W, YGR063C, YGR098C, YGR116W, YGR140W, YGR179C, YGR188C, YGR192C, YGR200C, YGR212W, YGR252W, YGR270W, YGR274C, YGR275W, YHL022C, YHR013C, YHR030C, YHR056C, YHR079C, YHR090C, YHR099W, YHR119W, YHR187W, YHR205W, YHR207C, YIL084C, YIL126W, YIL144W, YIL150C, YJL047C, YJL052W, YJL072C, YJL074C, YJL081C, YJL110C, YJL115W, YJL127C, YJL153C, YJL168C, YJL176C, YJL187C, YJL194W, YJR009C, YJR066W, YJR082C, YJR119C, YJR140C, YKL005C, YKL018W, YKL028W, YKL058W, YKL126W, YKR008W, YKR048C, YKR101W, YLL002W, YLL022C, YLR015W, YLR055C, YLR085C, YLR086W, YLR103C, YLR141W, YLR182W, YLR274W, YLR318W, YLR357W, YLR384C, YLR399C, YLR418C, YLR442C, YLR449W, YML010W, YML032C, YML041C, YML063W, YML069W, YML085C, YML102W, YML124C, YML127W, YMR020W, YMR033W, YMR037C, YMR044W, YMR048W, YMR075W, YMR076C, YMR127C, YMR176W, YMR223W, YMR236W, YMR263W, YMR284W, YMR312W, YNL021W, YNL031C, YNL088W, YNL097C, YNL107W, YNL112W, YNL132W, YNL136W, YNL167C, YNL206C, YNL236W, YNL246W, YNL273W, YNL312W, YNL330C, YNR023W, YOL004W, YOL006C, YOL012C, YOL051W, YOL054W, YOL068C, YOL069W, YOL146W, YOL148C, YOR001W, YOR023C, YOR025W, YOR026W, YOR038C, YOR061W, YOR064C, YOR073W, YOR123C, YOR151C, YOR194C, YOR213C, YOR244W, YOR253W, YOR290C, YOR304W, YPL001W, YPL015C, YPL016W, YPL018W, YPL042C, YPL047W, YPL082C, YPL086C, YPL101W, YPL116W, YPL127C, YPL129W, YPL138C, YPL153C, YPL181W, YPL209C, YPL235W, YPL240C, YPL254W, YPR018W, YPR019W, YPR023C, YPR031W, YPR034W, YPR068C, YPR086W, YPR133C, YPR135W, YPR161C.

S.10.3 `networkGWAS` (K_{lin}) on *H. sapiens* phenotype T2D

The nine identified neighborhoods, namely *ENSG00000152804*'s, *ENSG00000140718*'s, *ENSG00000148737*'s, *ENSG00000145996*'s, *ENSG00000109501*'s, *ENSG00000164756*'s, *ENSG00000134640*'s, *ENSG00000073792*'s and *ENSG00000187486*'s, represent a connected component composed of 144 genes:

Candidate genes:
ENSG00000004846, ENSG00000005339, ENSG00000014824, ENSG00000020633, ENSG00000047188, ENSG00000055130, ENSG00000065717, ENSG00000069431, ENSG00000070018, ENSG00000072062, ENSG00000072954, ENSG00000078369, ENSG00000079337, ENSG00000083896, ENSG00000087095, ENSG00000091428, ENSG00000091542, ENSG00000100219, ENSG00000100393, ENSG00000100601, ENSG00000103126, ENSG00000105856, ENSG00000105968, ENSG00000106829, ENSG00000110092, ENSG00000111640, ENSG00000112473, ENSG00000113558, ENSG00000114450, ENSG00000116128, ENSG00000121281, ENSG00000122873, ENSG00000124635, ENSG00000127955, ENSG00000129514, ENSG00000134982, ENSG00000136826, ENSG00000137760, ENSG00000138795, ENSG00000140332, ENSG00000140396, ENSG00000142875, ENSG00000143153, ENSG00000145362, ENSG00000145388, ENSG00000146457, ENSG00000149503, ENSG00000149658, ENSG00000152683, ENSG00000152977, ENSG00000154310, ENSG00000154342, ENSG00000155229, ENSG00000155897, ENSG00000157613, ENSG00000158161, ENSG00000158373, ENSG00000159217, ENSG00000159387, ENSG00000159692, ENSG00000161202, ENSG00000162337, ENSG00000164032, ENSG00000164944, ENSG00000165059, ENSG00000165323, ENSG00000165819, ENSG00000166167, ENSG00000166199, ENSG00000168036, ENSG00000168148, ENSG00000168646, ENSG00000170345, ENSG00000172197, ENSG00000172354, ENSG00000175029, ENSG00000177565, ENSG00000177606, ENSG00000180573, ENSG00000183207, ENSG00000184047, ENSG00000185130, ENSG00000185728, ENSG00000186174, ENSG00000187140, ENSG00000188486, ENSG00000189046, ENSG00000196591, ENSG00000196628, ENSG00000198492, ENSG00000198728, ENSG00000213676, ENSG00000234289, ENSG00000246705.

Candidate genes identified by the comparison partners, i.e., dmGWAS and NAGA:
ENSG00000104907, ENSG00000114353, ENSG00000116478, ENSG00000156052, ENSG00000173801, ENSG00000196367, ENSG00000196781, ENSG00000233822.

Genes involved in T2D-related processes:
ENSG00000006071, ENSG00000053918, ENSG00000054356, ENSG00000073792, ENSG00000101076, ENSG00000106633, ENSG00000109501, ENSG00000109919, ENSG00000115970, ENSG00000118217, ENSG00000120341, ENSG00000132170, ENSG00000134371, ENSG00000134640, ENSG00000136750, ENSG00000138160, ENSG00000140718, ENSG00000141646, ENSG00000142330, ENSG00000143842, ENSG00000145354, ENSG00000145996, ENSG00000147889, ENSG00000148737, ENSG00000149948, ENSG00000151353, ENSG00000151465, ENSG00000152254, ENSG00000152804, ENSG00000153814, ENSG00000153885, ENSG00000163281, ENSG00000163581, ENSG00000164756, ENSG00000168412, ENSG00000172260, ENSG00000173175, ENSG00000177508, ENSG00000178188, ENSG00000183049, ENSG00000187486, ENSG00000254647.

S.11 Figures

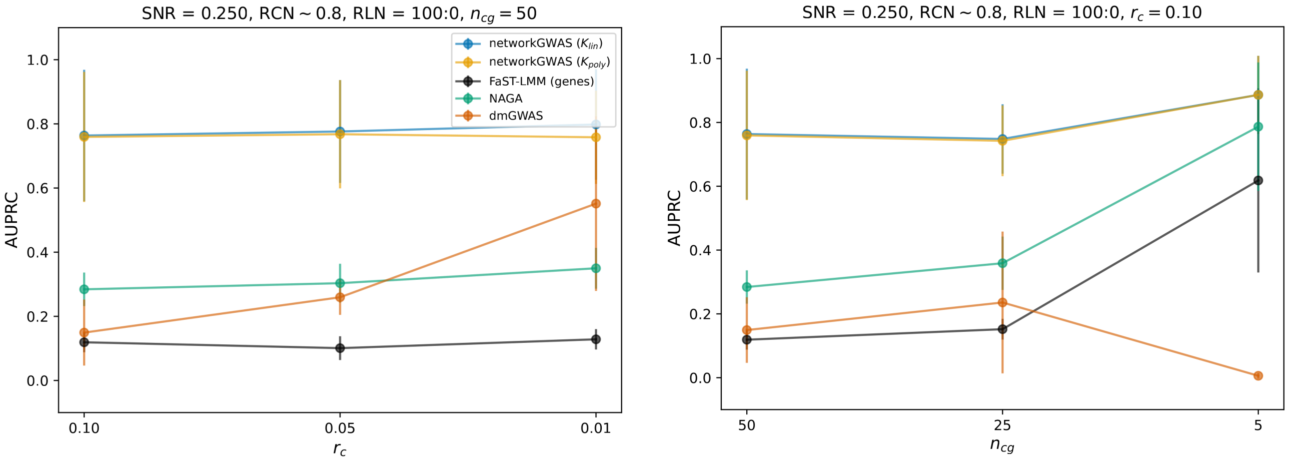

(a) (b)

Fig. S.1. Results from simulating the phenotypes of *A. thaliana*. We present results from our method (`networkGWAS`), using either a linear (K_{lin}) or a polynomial (K_{poly}) kernel, as well as the performance of other comparison methods. We show the average AUPRC when varying when varying the ratio of causal SNPs on a causal gene (r_c) (a) or the number of causal genes (n_{cg}) (b), while keeping the other four variables fixed.

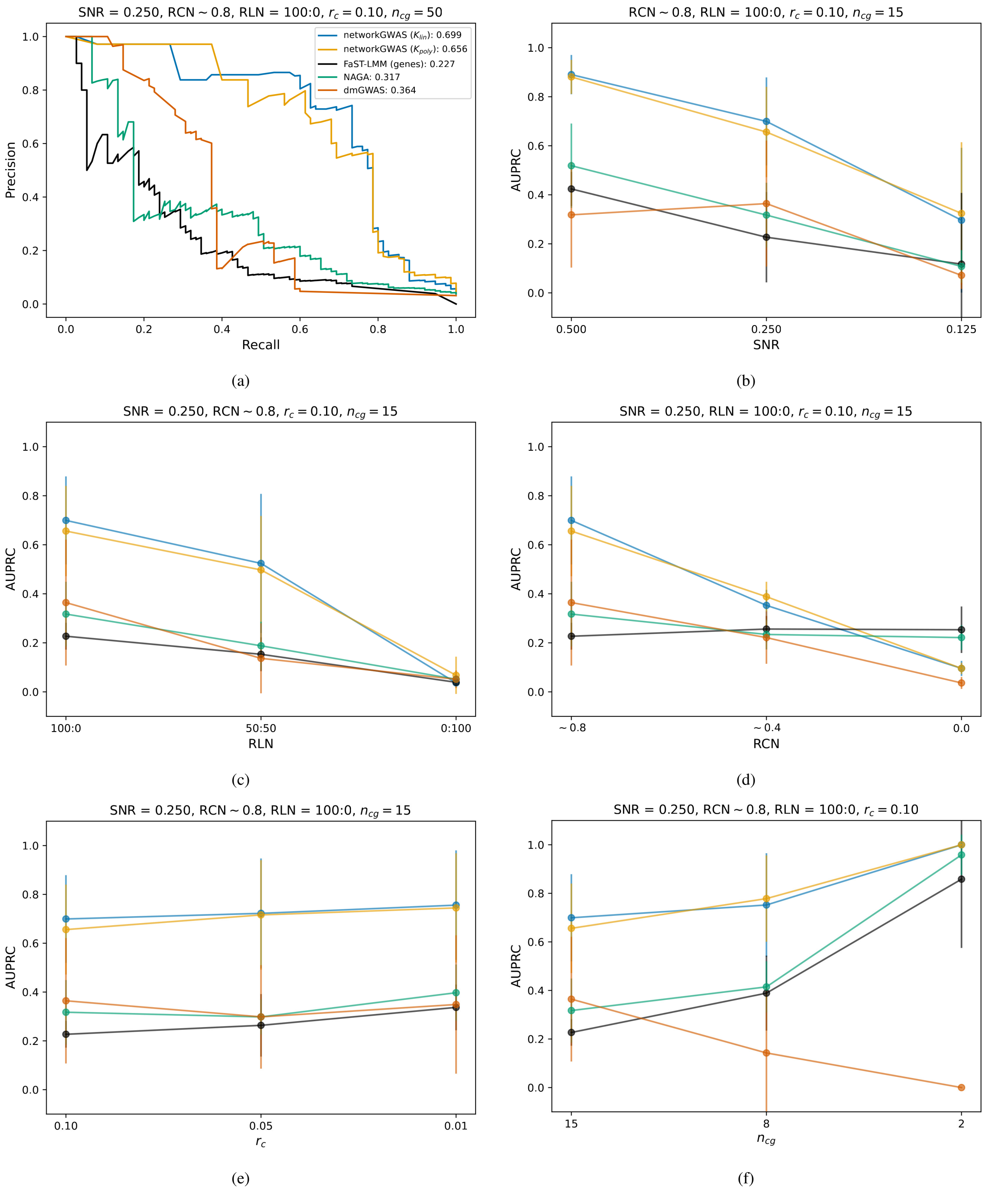

Fig. S.2. Results from our simulated rare variants use case. We present results from our method (`networkGWAS`), using either a linear (K_{lin}) or a polynomial (K_{poly}) kernel, as well as the performance of other comparison methods. In Subfigure S2a, we show the AUPRC of the baseline scenario (S0). In Subfigures 3-b-3d, we vary one variable while keeping the other four fixed: the signal-to-noise ratio (SNR) (S2b), the mixing ratio of linear-to-nonlinear (RLN) signal (S2c), the mean ratio of causal neighbors (RCN) (S2d), the ratio of causal SNPs on a causal gene (r_c) (S2e), and the number of causal genes (n_{cg}) (S2f).

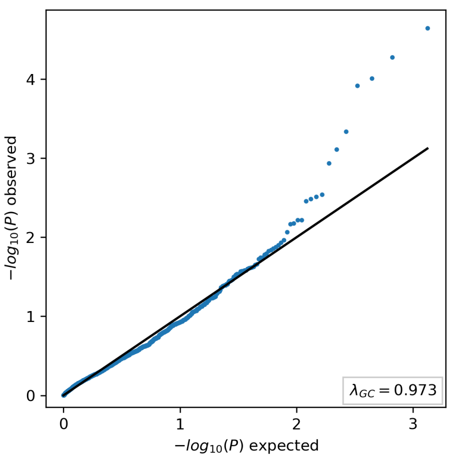

(a)

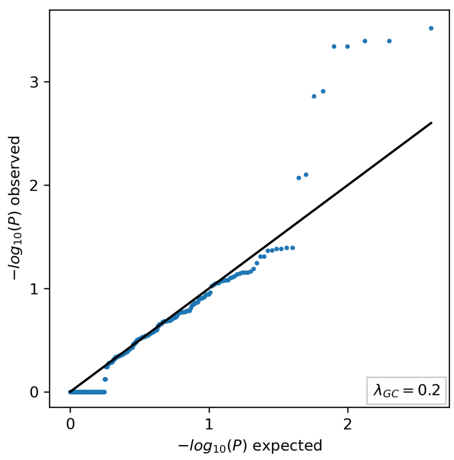

(b)

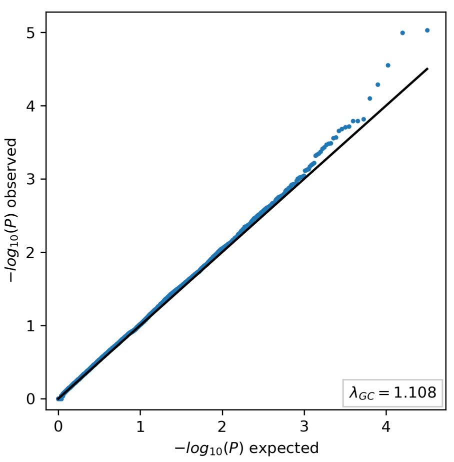

(c)

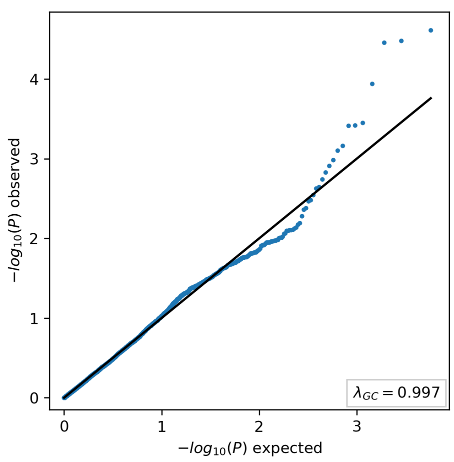

(d)

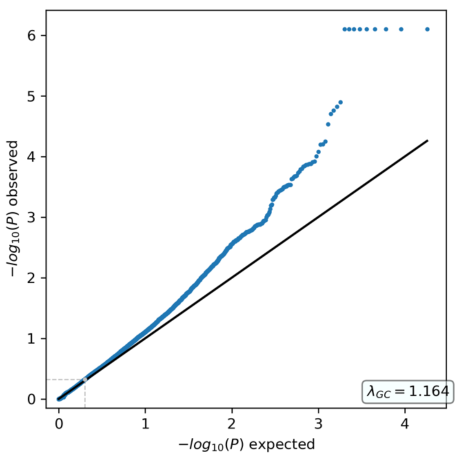

(e)

Fig. S.3. Distributions of the p-values obtained using `networkGWAS` on different use cases: (a) a scenario from the *A. thaliana* semi-simulations, setting S5; (b) a scenario from setting S0 for *H. sapiens* synthetic simulations; (c) the *A. thaliana* phenotype 524; (d) the *S. cerevisiae* phenotype YPGALACTOSE; (e) the T2D phenotype form the Estonian BioBank data.

S.12 Tables

Organism	Network	#nodes	#edges	#samples	Permutation time [s]	LMM time [s/m/h]
A. thaliana (semi-simulations)	TAIR [18]	1,327	3,236	2,029	$\sim$15 s	$\sim$1 m
A. thaliana	STRING [35]	31,568	825,834	512 to 904	$\sim$ 30 m	$\sim$8 h
S. cerevisiae	STRING [35]	5,737	208,414	971	$\sim$5 m	$\sim$10 m
H. sapiens (simulations)	STRING [35]	308	1,180	500	$\sim$ 5 s	$\sim$30 s
H. sapiens	STRING [35]	18,048	403,562	136,772 to 199,466	$\sim$ 15 m	$\sim$12 h

Table S.1. Overview of the PPI networks sizes and samples sizes across our simulated settings and applications. We furthermore report their respective running times. The column "permutation time" reports the time required to perform *one* permutation (i.e., circular and degree-preserving network permutation), while the column named "LMM time" refers to the time required to obtain the lrt statistics per each neighborhood on *one* setting, permuted or non-permuted. The reported results are for K_{lin} networkGWAS. Calculating the total running time depends on the available resources and the possibility to run multiple jobs in parallel. In the worst case scenario of having to run all the permutations sequentially, to calculate the total running time one has to sum "permutation time" and "LMM time" and multiply the obtained result by the total number of permutations. These results are obtained on a server equipped with Intel Xeon Gold 6254 CPU and 1TB of RAM.

	S0	S1	S2	S3	S4	S5	S6	S7	S8	S9	S10
**Signal-to-noise ratio (SNR)**	0.250	0.125	0.500	0.250	0.250	0.250	0.250	0.250	0.250	0.250	0.250
**Ratio of linear to non-linear (RLN)**	100 : 0	100 : 0	100 : 0	50 : 50	0 : 100	100 : 0	100 : 0	100 : 0	100 : 0	100 : 0	100 : 0
**Ratio of causal neighbors (RCN)**	0.8	0.8	0.8	0.8	0.8	0.4	0.0	0.8	0.8	0.8	0.8
**Ratio of causal SNPs (r_c)**	0.10	0.10	0.10	0.10	0.10	0.10	0.10	0.05	0.01	0.10	0.10
**Number of causal genes (n_{cg})**	50	50	50	50	50	50	50	50	50	25	5

Table S.2. Overview of simulation settings for artificial phenotypes. All the parameters, i.e., the signal-to-noise ratio (SNR), the mixing ratio of linear to non-linear signal (RLN), the ratio of causal neighbors (RCN), the ratio of causal SNPs (r_c), and the number of causal genes (n_{cg}) are systematically varied.

	id	phenotype	cohort size n	original study
**N01**	518	relative lifetime fitness in Spain for high precipitation treatment and low density	512	[10]
**N02**	519	relative lifetime fitness in Spain for high precipitation treatment and high density	512	[10]
**N03**	522	relative lifetime fitness in Germany for high precipitation treatment and low density	512	[10]
**N04**	523	relative lifetime fitness in Germany for high precipitation treatment and high density	512	[10]
**N05**	524	relative lifetime fitness in Germany for low precipitation treatment and low density	512	[10]
**N06**	534	relative seed production in Spain for high precipitation treatment and low density	511	[10]
**N07**	535	relative seed production in Spain for high precipitation treatment and high density	512	[10]
**N08**	538	relative seed production in Germany for high precipitation treatment and low density	512	[10]
**N09**	539	relative seed production in Germany for high precipitation treatment and high density	511	[10]
**N10**	700	length of main flowering stem	680	[37]
**N11**	704	rosette leaf number	850	[37]
**N12**	705	cauline leaf number	904	[37]
**N13**	707	diameter of rosette	656	[37]

Table S.3. Overview of *A. thaliana* phenotypes.

id	media composition	temperature
YPACETATE	2% bactopeptone; 1% yeast extract; 2% acetate; 2% agar	30°C
YPD14	2% bactopeptone; 1% yeast extract; 2% glucose; 2% agar	14°C
YPD40	2% bactopeptone; 1% yeast extract; 2% glucose; 2% agar	40°C
YPD42	2% bactopeptone; 1% yeast extract; 2% glucose; 2% agar	42°C
YPDANISO50	2% bactopeptone; 1% yeast extract; 2% glucose; 2% agar; anisomycin 50 µg/ml	30°C
YPDBENOMYL200	2% bactopeptone; 1% yeast extract; 2% glucose; 2% agar; benomyl 200 µg/ml	30°C
YPDCHX05	2% bactopeptone; 1% yeast extract; 2% glucose; 2% agar; cycloheximide 0.5 µg/ml	30°C
YPDDMSO	2% bactopeptone; 1% yeast extract; 2% glucose; 2% agar; DMSO 6%	30°C
YPDETOH	2% bactopeptone; 1% yeast extract; 2% glucose; 2% agar; ethanol 15%	30°C
YPDFLUCONAZOLE	2% bactopeptone; 1% yeast extract; 2% glucose; 2% agar; fluconazole 20 µg/ml	30°C
YPDFORMAMIDE4	2% bactopeptone; 1% yeast extract; 2% glucose; 2% agar; formamide 4%	30°C
YPDFORMAMIDE5	2% bactopeptone; 1% yeast extract; 2% glucose; 2% agar; formamide 5%	30°C
YPDHU	2% bactopeptone; 1% yeast extract; 2% glucose; 2% agar; hydroxyurea 30 mg/ml	30°C
YPDLICL250MM	2% bactopeptone; 1% yeast extract; 2% glucose; 2% agar; LiCl 250 mM	30°C
YPDMV	2% bactopeptone; 1% yeast extract; 2% glucose; 2% agar; methylviologen 20 mM	30°C
YPDNACL1M	2% bactopeptone; 1% yeast extract; 2% glucose; 2% agar; NaCl 1M	30°C
YPDNACL15M	2% bactopeptone; 1% yeast extract; 2% glucose; 2% agar; NaCl 1.5M	30°C
YPETHANOL	2% bactopeptone; 1% yeast extract; 2% ethanol; 2% agar	30°C
YPGALACTOSE	2% bactopeptone; 1% yeast extract; 2% galactose; 2% agar	30°C
YPGLYCEROL	2% bactopeptone; 1% yeast extract; 2% glycerol; 2% agar	30°C
YPRIBOSE	2% bactopeptone; 1% yeast extract; 2% ribose; 2% agar	30°C
YPSORBITOL	2% bactopeptone; 1% yeast extract; 2% sorbitol; 2% agar	30°C
YPXYLOSE	2% bactopeptone; 1% yeast extract; 2% xylose; 2% agar	30°C

Table S.4. Overview of *S. cerevisiae* phenotypes. The phenotypes are calculated as the growth ratio between the listed stress conditions and the standard growing condition for *S. cerevisiae*, namely a medium composed of 2% bactopeptone, 1% yeast extract, 2% glucose, and 2% agar, at 30°C.

id	univariate GWAS	original study [25]
YPACETATE	15S_rRNA_2	-
YPD14	-	-
YPD40	-	-
YPD42	Q0045	-
YPDANISO50	YER180C, YHR164C, YIL147C, YNL205C, YOR009W, YOR127W, YOR138C	-
YPDBENOMYL200	YLR395C	YLR395C
YPDCHX05	-	-
YPDDMSO	-	-
YPDETOH	-	-
YPDFLUCONAZOLE	-	-
YPDFORMAMIDE4	-	-
YPDFORMAMIDE5	-	-
YPDHU	YDL056W	YDL056W
YPDLICL250MM	YER119C, YGR249W, YML094W, Q0050, Q0065, Q0045	-
YPDMV	-	-
YPDNACL1M	YPR083W	YPR083W
YPDNACL15M	-	-
YPETHANOL	-	-
YPGALACTOSE	-	YMR163C, YKR094C - YKR095W; YLR423C - YLR424W
YPGLYCEROL	YLR006C, Q0045	YLR006C
YPRIBOSE	-	YLR006C; YIL052C - YIL051C
YPSORBITOL	YDR096W, YLR006C	YDR096W, YLR006C
YPXYLOSE	-	-

Table S.5. Overview of results obtained with the univariate GWAS analyses on the *S. cerevisiae* phenotypes. The column named “univariate GWAS" refers to the analysis we performed, while “original study" refers to the single SNP GWAS analysis done by Peter *et al.*. We report the genes onto which the statistically associated SNPs can be positionally mapped. As appreciable from the table, there is consistency for five phenotypes. The witnessed differences may be due to differing SNPs search spaces and type of multiple testing correction used.

chromosome	SNP	position	gene
2	rs1537	52083	YBL088C
2	rs1555	55192	YBL088C
2	rs2081	121589	YBL052C
2	rs3760	404662	YBR081C
4	rs16176	1137328	YDR334W
4	rs16184	1138235	YDR334W
4	rs18032	1412942	YDR477W
5	rs21237	379893	YER110C
5	rs21243	381575	YER110C
5	rs21246	381978	YER110C
7	rs25594	194505	YGL163C
7	rs27623	480515	YGL008C
10	rs38621	70181	YJL194W
10	rs42210	559813	YJR066W
10	rs42233	562628	YJR066W
10	rs42239	562775	YJR066W
10	rs42885	645972	YJR119C
10	rs42886	646155	YJR119C
12	rs55469	957510	YLR418C
12	rs55977	1020763	YLR442C
13	rs56319	23620	YML124C
13	rs58911	414299	YMR075W
14	rs62671	18355	YNL330C
14	rs62977	48880	YNL312W
14	rs64341	207220	YNL236W
14	rs64691	258013	YNL206C
14	rs66378	461930	YNL088W
15	rs71312	378902	YOR025W
15	rs71489	402926	YOR038C
15	rs73162	616666	YOR151C
15	rs75222	884597	YOR304W
16	rs78517	263119	YPL153C

Table S.6. Subset of the SNPs identified by *post hoc* Lasso analysis on the SNPs included in the two statistically associated neighborhoods found by networkGWAS (K_{lin}) on the natural *S. cerevisiae* phenotype YPGALACTOSE.

picture(0,0)(-35,0)(1,0)30 (0,35)(0,-1)30 picture picture(0,0)(35,0)(-1,0)30 (0,35)(0,-1)30 picture

References in the supplementary

[S1]Regulation of transforming growth factor β in diabetic nephropathy: Implications for treatment. *Seminars in Nephrology*, 27(2):153–160, 2007.

[S2]A. Auton et al. A global reference for human genetic variation. *Nature*, 526(7571):68–74, 2015.

[S3]Y. Benjamini and Y. Hochberg. Controlling the false discovery rate: a practical and powerful approach to multiple testing. *J. R. Stat. Soc., B: Stat. Methodol.*, 57(1):289–300, 1995.

[S4]N. E. Breslow and D. G. Clayton. Approximate inference in generalized linear mixed models. *J. Am. Stat. Assoc.*, 88:9, 1993.

[S5]J. Chen et al. Role of Wnt signaling pathways in type 2 diabetes mellitus. *Mol. Cell. Biochem.*, 476:2219–2232, 2021.

[S6]J. M. Cherry et al. Genetic and physical maps of *Saccharomyces cerevisiae*. *Nature*, 387:67–73, 1997.

[S7]P. Danecek et al. Twelve years of samtools and bcftools. *Gigascience*, 10(2):giab008, 2021.

[S8]A. Dimitromanolakis et al. sim1000g: a user-friendly genetic variant simulator in r for unrelated individuals and family-based designs. *BMC Bioinf.*, 20(26), 2019.

[S9]B. Efron. Simultaneous inference: When should hypothesis testing problems be combined? *Ann Appl Stat*, 2(1):197 – 223, 2008.

[S10]M. Exposito-Alonso et al. Natural selection on the arabidopsis thaliana genome in present and future climates. *Nature*, 2019.

[S11]A. Frankish et al. GENCODE 2021. *Nucleic Acids Research*, 49(D1):D916–D923, 2020.

[S12]M. E. Goddard et al. Estimating effects and making predictions from genome-wide marker data. *Stat Sci*, 24(4):517–529, 2009.

[S13]S. Greven et al. Restricted Likelihood Ratio Testing for Zero Variance Components in Linear Mixed Models. *J. Comput. Graph. Statist.*, 17(4):870–891, 2008.

[S14]A. Hamosh et al. Online Mendelian Inheritance in Man (OMIM). *Human mutation*, 15(1):57–61, 2000.

[S15]B. J. Hayes, P. M. Visscher, and M. E. Goddard. Increased accuracy of artificial selection by using the realized relationship matrix. *Genet. Res.*, 91(1):47–60, 2009.

[S16]J. Joo et al. Multiple testing correction in linear mixed models. *Genome Biol.*, 17(62), 2016.

[S17]J. Jourquin et al. GLAD4U: deriving and prioritizing gene lists from PubMed literature. *BMC genomics*, 13(8):S20, 2012.

[S18]P. Lamesch et al. The Arabidopsis information resource (TAIR): improved gene annotation and new tools. *Nucleic Acids Res.*, 40(D1):D1202–D1210, 2012.

[S19]L. Leitsalu et al. Cohort Profile: Estonian Biobank of the Estonian Genome Center, University of Tartu. *Int. J. Epidemiol.*, 44(4):1137–1147, 2014.

[S20]S. Li Calzi et al. Endothelial progenitor dysfunction in the pathogenesis of diabetic retinopathy: treatment concept to correct diabetes-associated deficits. *EPMA J.*, 1:88–100, 2010.

[S21]C. Lippert et al. FaST linear mixed models for genome-wide association studies. *Nat Methods*, 8(10):833–835, 2011.

[S22]C. Lippert et al. Greater power and computational efficiency for kernel-based association testing of sets of genetic variants. *Bioinf.*, 30(22):3206–3214, 2014.

[S23]J. Listgarten et al. A powerful and efficient set test for genetic markers that handles confounders. *Bioinf.*, 29(12):1526–1533, 2013.

[S24]H. Mi et al. Panther version 7: improved phylogenetic trees, orthologs and collaboration with the gene ontology consortium. *Nucleic Acids Res.*, 38:D204–D210, 2009.

[S25]J. Peter and others. Genome evolution across 1,011 Saccharomyces cerevisiae isolates. *Nature*, 556:339—-344, 2018.

[S26]C. B. Peterson et al. Many Phenotypes Without Many False Discoveries: Error Controlling Strategies for Multitrait Association Studies. *Genet. Epidemiol.*, 40(1):45–56, 2016.

[S27]J. C. Pinheiro and D. M. Bates. Linear Mixed-Effects Models: Basic Concepts and Examples. In *Mixed-Effects Models in S and S-PLUS*, Statistics and Computing. Springer, New York, NY, 2000.

[S28]J. Piñero et al. The DisGeNET knowledge platform for disease genomics: 2019 update. *Nucleic Acids Res.*, 48(D1):D845–D855, 2019.

[S29]A. L. Price et al. Principal components analysis corrects for stratification in genome-wide association studies. *Nat Genet*, 38(8):904–909, 2006.

[S30]S. Purcell et al. Plink: a toolset for whole-genome association and population-based linkage analysis. *Am. J. Hum. Genet.*, 81, 2007.

[S31]S. K. Sarkar and C.-K. Chang. The simes method for multiple hypothesis testing with positively dependent test statistics. *JASA*, 92(440):1601–1608, 1997.

[S32]Ü. Seren et al. AraPheno: a public database for Arabidopsis thaliana phenotypes. *Nucleic Acids Res.*, 45(D1):D1054–D1059, 2016.

[S33]Shaun Purcell. PLINK 2.0. *https://www.cog-genomics.org/plink/2.0/*, 2007.

[S34]R. J. Simes. An improved Bonferroni procedure for multiple tests of significance. *Biometrika*, 73(3):751–754, 1986.

[S35]D. Szklarczyk et al. The STRING database in 2021: customizable protein–protein networks, and functional characterization of user-uploaded gene/measurement sets. *Nucleic Acids Res.*, 49(D1):D605–D612, 2021.

[S36]TAIR. GO Term Enrichment for Plants. *https://www.arabidopsis.org/tools/go_term_enrichment.jsp*, 2021.

[S37]The 1001 Genomes Consortium. 1,135 genomes reveal the global pattern of polymorphism in arabidopsis thaliana. *Cell*, 166(2), 2016.

[S38]P. D. Thomas et al. Panther: A library of protein families and subfamilies indexed by function. *Genome Res.*, 13:2129–2141, 2003.

[S39]M. Togninalli et al. The AraGWAS Catalog: a curated and standardized Arabidopsis thaliana GWAS catalog. *Nucleic Acids Res.*, 46(D1):D1150–D1156, 2018.

[S40]J. Wang et al. Web-based gene set analysis toolkit (webgestalt): update 2013. *Nucleic acids res.*, 41(W1):W77–W83, 2013.

[S41]S. S. Wilks. The Large-Sample Distribution of the Likelihood Ratio for Testing Composite Hypotheses. *Ann. Math. Stat.*, 9(1):60–62, 1938.

[S42]C. Wu, I. MacLeod, and A. I. Su. BioGPS and MyGene.info: organizing online, gene-centric information. *Nucleic Acids Res.*, 41(D1):D561–D565, 2013.

[S43]J. Xin et al. High-performance web services for querying gene and variant annotation. *Genome Biol.*, 17(1):1–7, 2016.

picture(0,0)(-35,0)(1,0)30 (0,-35)(0,1)30 picture picture(0,0)(35,0)(-1,0)30 (0,-35)(0,1)30 picture
