# Supplementary material for: networkGWAS: a network-based approach to discover genetic associations
Source: Bioinformatics. 2023 Jun 7;39(6):btad370. doi: 10.1093/bioinformatics/btad370 (PMC10281858; doi:10.1093/bioinformatics/btad370)
Supplement: btad370_Supplementary_Data [file btad370_supplementary_data.zip › enrichment_results/enrichment_results/S_cerevisiae_enrichment.pdf]

PANTHER17.0 Released.

Analysis Summary: Please report in publication

Analysis Type: PANTHER Overrepresentation Test (Released 20220202)

Annotation Version and Release Date: PANTHER version 17.0 Released 2022-02-22

Analyzed List: ass_neighborhood.txt (Saccharomyces cerevisiae) Change

Reference List: Saccharomyces cerevisiae (all genes in database) Change

Annotation Data Set: PANTHER GO-Slim Biological Process

Test Type: Fisher's Exact Binomial

Correction: Calculate False Discovery Rate Use the Bonferroni correction for multiple testing No correction

Results

	Reference list	ass_neighborhood.txt
Uniquely Mapped IDS:	6050 out of 6050	248 out of 248
Unmapped IDs:	0	17
Multiple mapping information:	0	0

Export Table XML with user input ids JSON with user input ids View: -- Please select a chart to display --

Displaying only results for FDR P < 0.05, click here to display all results

	Saccharomyces cerevisiae (REF)	ass_neighborhood.txt (Hierarchy NEW!)					
PANTHER GO-Slim Biological Process	#	#	expected	Fold Enrichment	+/-	raw P value	FDR
histone deacetylation	5	5	.20	24.40	+	1.95E-05	1.89E-04
↳protein deacetylation	5	5	.20	24.40	+	1.95E-05	1.87E-04
↳protein deacylation	5	5	.20	24.40	+	1.95E-05	1.88E-04
↳macromolecule deacylation	5	5	.20	24.40	+	1.95E-05	1.85E-04
↳macromolecule modification	441	44	18.08	2.43	+	1.27E-07	1.59E-06
↳macromolecule metabolic process	1316	132	53.95	2.45	+	4.96E-26	1.88E-24
↳organic substance metabolic process	1774	136	72.72	1.87	+	3.27E-16	8.73E-15
↳metabolic process	1829	137	74.97	1.83	+	1.96E-15	5.03E-14
↳cellular protein modification process	390	40	15.99	2.50	+	2.17E-07	2.66E-06
↳protein modification process	390	40	15.99	2.50	+	2.17E-07	2.64E-06
↳protein metabolic process	664	42	27.22	1.54	+	5.38E-03	3.27E-02
↳nitrogen compound metabolic process	1566	131	64.19	2.04	+	1.31E-18	3.70E-17
↳primary metabolic process	1661	132	68.09	1.94	+	7.84E-17	2.13E-15
↳cellular protein metabolic process	637	42	26.11	1.61	+	3.20E-03	2.08E-02
↳cellular macromolecule metabolic process	1056	124	43.29	2.86	+	4.77E-30	4.33E-28
↳cellular metabolic process	1751	133	71.78	1.85	+	2.87E-15	7.23E-14
↳cellular process	2615	169	107.19	1.58	+	1.25E-14	3.05E-13
↳histone modification	38	29	1.56	18.62	+	7.57E-24	2.64E-22
DNA replication-independent chromatin assembly	5	5	.20	24.40	+	1.95E-05	1.84E-04
↳DNA replication-independent chromatin organization	6	6	.25	24.40	+	2.66E-06	2.78E-05
↳chromatin organization	51	35	2.09	16.74	+	1.63E-27	8.23E-26
↳cellular component organization	658	73	26.97	2.71	+	6.80E-15	1.68E-13
↳cellular component organization or biogenesis	805	73	33.00	2.21	+	1.15E-10	2.00E-09
↳chromosome organization	126	51	5.16	9.87	+	1.94E-31	2.03E-29
↳organelle organization	532	66	21.81	3.03	+	1.49E-15	3.89E-14

↳chromatin assembly	19	14	.78	17.98	+	6.29E-12	1.17E-10
↳DNA packaging	23	15	.94	15.91	+	3.84E-12	7.26E-11
↳DNA conformation change	30	18	1.23	14.64	+	7.15E-14	1.54E-12
↳chromatin assembly or disassembly	19	14	.78	17.98	+	6.29E-12	1.16E-10
↳cellular component assembly	222	26	9.10	2.86	+	3.90E-06	4.02E-05
DNA unwinding involved in DNA replication	3	3	.12	24.40	+	1.10E-03	7.83E-03
↳DNA duplex unwinding	7	3	.29	10.46	+	5.89E-03	3.55E-02
↳DNA geometric change	7	3	.29	10.46	+	5.89E-03	3.56E-02
↳DNA-dependent DNA replication	42	14	1.72	8.13	+	1.98E-08	2.93E-07
↳DNA replication	44	14	1.80	7.76	+	3.22E-08	4.47E-07
↳cellular macromolecule biosynthetic process	592	92	24.27	3.79	+	5.69E-29	3.52E-27
↳macromolecule biosynthetic process	598	92	24.51	3.75	+	1.14E-28	6.74E-27
↳organic substance biosynthetic process	888	95	36.40	2.61	+	7.99E-19	2.37E-17
↳biosynthetic process	890	95	36.48	2.60	+	8.73E-19	2.53E-17
↳cellular biosynthetic process	872	94	35.74	2.63	+	6.20E-19	1.92E-17
histone H3 acetylation	3	3	.12	24.40	+	1.10E-03	7.79E-03
↳histone acetylation	19	16	.78	20.54	+	4.31E-14	1.01E-12
↳internal peptidyl-lysine acetylation	19	16	.78	20.54	+	4.31E-14	9.61E-13
↳internal protein amino acid acetylation	19	16	.78	20.54	+	4.31E-14	9.93E-13
↳protein acetylation	24	16	.98	16.26	+	5.59E-13	1.12E-11
↳protein acylation	33	16	1.35	11.83	+	2.16E-11	3.93E-10
↳peptidyl-lysine acetylation	19	16	.78	20.54	+	4.31E-14	9.77E-13
↳peptidyl-lysine modification	48	21	1.97	10.67	+	6.82E-14	1.50E-12
↳peptidyl-amino acid modification	93	25	3.81	6.56	+	2.31E-12	4.43E-11
nucleosome assembly	6	5	.25	20.33	+	3.46E-05	3.18E-04
↳nucleosome organization	21	15	.86	17.43	+	1.48E-12	2.93E-11
↳chromatin remodeling	41	28	1.68	16.66	+	4.12E-22	1.33E-20
↳protein-DNA complex subunit organization	45	25	1.84	13.55	+	3.07E-18	8.53E-17
↳protein-containing complex subunit organization	193	28	7.91	3.54	+	2.95E-08	4.18E-07
↳protein-DNA complex assembly	29	14	1.19	11.78	+	4.23E-10	7.02E-09
↳cellular protein-containing complex assembly	156	17	6.39	2.66	+	4.27E-04	3.40E-03
↳protein-containing complex assembly	161	17	6.60	2.58	+	5.95E-04	4.50E-03
double-strand break repair via break-induced replication	11	9	.45	19.96	+	2.27E-08	3.28E-07
↳double-strand break repair via homologous recombination	32	13	1.31	9.91	+	9.49E-09	1.47E-07
↳recombinational repair	35	13	1.43	9.06	+	2.26E-08	3.30E-07
↳DNA recombination	52	15	2.13	7.04	+	3.08E-08	4.32E-07
↳DNA metabolic process	141	32	5.78	5.54	+	8.78E-14	1.87E-12
↳nucleic acid metabolic process	711	110	29.15	3.77	+	1.29E-35	2.19E-33
↳nucleobase-containing compound metabolic process	841	112	34.47	3.25	+	7.00E-31	6.80E-29
↳cellular nitrogen compound metabolic process	1032	112	42.30	2.65	+	1.24E-23	4.20E-22
↳organic cyclic compound metabolic process	916	112	37.55	2.98	+	8.30E-28	4.34E-26
↳heterocycle metabolic process	896	112	36.73	3.05	+	1.35E-28	7.66E-27
↳cellular aromatic compound metabolic process	896	112	36.73	3.05	+	1.35E-28	7.35E-27
↳DNA repair	98	21	4.02	5.23	+	5.01E-09	8.02E-08
↳cellular response to DNA damage stimulus	109	27	4.47	6.04	+	1.51E-12	2.94E-11
↳cellular response to stress	200	30	8.20	3.66	+	4.52E-09	7.32E-08
↳response to stress	205	30	8.40	3.57	+	7.56E-09	1.18E-07
↳response to stimulus	352	41	14.43	2.84	+	5.09E-09	8.06E-08
↳cellular response to stimulus	343	41	14.06	2.92	+	2.55E-09	4.18E-08
↳double-strand break repair	42	14	1.72	8.13	+	1.98E-08	2.96E-07
positive regulation of transcription elongation from RNA polymerase II promoter	5	4	.20	19.52	+	2.53E-04	2.12E-03
↳positive regulation of transcription by RNA polymerase II	57	16	2.34	6.85	+	1.46E-08	2.20E-07
↳regulation of transcription by RNA polymerase II	175	52	7.17	7.25	+	9.72E-27	4.14E-25

↳regulation of transcription, DNA-templated	219	63	8.98	7.02	+	6.74E-32	9.17E-30
↳regulation of nucleic acid-templated transcription	219	63	8.98	7.02	+	6.74E-32	8.34E-30
↳regulation of RNA biosynthetic process	219	63	8.98	7.02	+	6.74E-32	7.64E-30
↳regulation of RNA metabolic process	248	63	10.17	6.20	+	2.80E-29	1.82E-27
↳regulation of macromolecule metabolic process	378	72	15.49	4.65	+	8.06E-27	3.54E-25
↳regulation of metabolic process	392	73	16.07	4.54	+	1.11E-26	4.59E-25
↳regulation of biological process	576	87	23.61	3.68	+	2.19E-26	8.52E-25
↳biological regulation	670	89	27.46	3.24	+	1.96E-23	6.52E-22
↳regulation of nucleobase-containing compound metabolic process	252	64	10.33	6.20	+	9.25E-30	6.99E-28
↳regulation of nitrogen compound metabolic process	325	65	13.32	4.88	+	4.25E-25	1.56E-23
↳regulation of cellular metabolic process	343	69	14.06	4.91	+	7.25E-27	3.29E-25
↳regulation of cellular process	530	84	21.73	3.87	+	1.14E-26	4.57E-25
↳regulation of primary metabolic process	334	65	13.69	4.75	+	1.62E-24	5.81E-23
↳regulation of macromolecule biosynthetic process	271	66	11.11	5.94	+	8.17E-30	6.54E-28
↳regulation of biosynthetic process	274	66	11.23	5.88	+	1.43E-29	1.02E-27
↳regulation of cellular biosynthetic process	274	66	11.23	5.88	+	1.43E-29	9.71E-28
↳regulation of gene expression	313	67	12.83	5.22	+	2.05E-27	9.95E-26
↳regulation of cellular macromolecule biosynthetic process	269	66	11.03	5.99	+	5.61E-30	4.77E-28
↳positive regulation of transcription, DNA-templated	73	17	2.99	5.68	+	5.42E-08	7.23E-07
↳positive regulation of nucleic acid-templated transcription	73	17	2.99	5.68	+	5.42E-08	7.30E-07
↳positive regulation of RNA biosynthetic process	73	17	2.99	5.68	+	5.42E-08	7.16E-07
↳positive regulation of RNA metabolic process	93	17	3.81	4.46	+	1.10E-06	1.23E-05
↳positive regulation of nucleobase-containing compound metabolic process	94	17	3.85	4.41	+	1.26E-06	1.38E-05
↳positive regulation of cellular metabolic process	126	20	5.16	3.87	+	8.95E-07	1.01E-05
↳positive regulation of metabolic process	129	20	5.29	3.78	+	1.24E-06	1.38E-05
↳positive regulation of biological process	146	20	5.98	3.34	+	6.82E-06	6.82E-05
↳positive regulation of cellular process	143	20	5.86	3.41	+	5.15E-06	5.23E-05
↳positive regulation of nitrogen compound metabolic process	120	19	4.92	3.86	+	1.75E-06	1.89E-05
↳positive regulation of macromolecule metabolic process	124	20	5.08	3.93	+	7.15E-07	8.11E-06
↳positive regulation of macromolecule biosynthetic process	85	18	3.48	5.17	+	7.66E-08	9.84E-07
↳positive regulation of biosynthetic process	86	18	3.53	5.11	+	8.94E-08	1.14E-06
↳positive regulation of cellular biosynthetic process	86	18	3.53	5.11	+	8.94E-08	1.13E-06
↳positive regulation of DNA-templated transcription, elongation	8	4	.33	12.20	+	9.05E-04	6.58E-03
↳regulation of DNA-templated transcription, elongation	9	5	.37	13.55	+	1.36E-04	1.17E-03
↳regulation of transcription elongation from RNA polymerase II promoter	6	5	.25	20.33	+	3.46E-05	3.20E-04
histone H4 acetylation	5	4	.20	19.52	+	2.53E-04	2.11E-03
regulation of DNA replication	4	3	.16	18.30	+	1.88E-03	1.28E-02
mitotic DNA replication initiation	4	3	.16	18.30	+	1.88E-03	1.28E-02
↳mitotic DNA replication	8	6	.33	18.30	+	8.08E-06	8.03E-05
↳nuclear DNA replication	10	7	.41	17.08	+	1.87E-06	1.99E-05
↳cell cycle DNA replication	10	7	.41	17.08	+	1.87E-06	2.01E-05
↳cell cycle process	143	33	5.86	5.63	+	2.30E-14	5.48E-13
↳cell cycle	155	33	6.35	5.19	+	1.66E-13	3.38E-12
↳mitotic cell cycle process	101	23	4.14	5.56	+	3.18E-10	5.40E-09
↳mitotic cell cycle	101	23	4.14	5.56	+	3.18E-10	5.47E-09
↳DNA replication initiation	17	8	.70	11.48	+	3.12E-06	3.24E-05
histone H3-K4 methylation	6	4	.25	16.26	+	4.09E-04	3.31E-03
↳histone lysine methylation	8	5	.33	15.25	+	9.03E-05	8.03E-04
↳histone methylation	11	6	.45	13.31	+	3.01E-05	2.83E-04
↳protein methylation	20	6	.82	7.32	+	4.16E-04	3.35E-03
↳protein alkylation	20	6	.82	7.32	+	4.16E-04	3.33E-03
↳peptidyl-lysine methylation	15	5	.61	8.13	+	8.67E-04	6.41E-03
histone exchange	7	4	.29	13.94	+	6.22E-04	4.68E-03

RNA polymerase II preinitiation complex assembly	9	5	.37	13.55	+	1.36E-04	1.18E-03
↳transcription initiation from RNA polymerase II promoter	19	8	.78	10.27	+	5.98E-06	6.03E-05
↳DNA-templated transcription, initiation	28	10	1.15	8.71	+	1.34E-06	1.45E-05
↳transcription, DNA-templated	263	77	10.78	7.14	+	6.10E-40	2.77E-37
↳nucleic acid-templated transcription	263	77	10.78	7.14	+	6.10E-40	4.15E-37
↳RNA biosynthetic process	270	77	11.07	6.96	+	2.94E-39	1.00E-36
↳RNA metabolic process	592	81	24.27	3.34	+	7.94E-22	2.51E-20
↳nucleobase-containing compound biosynthetic process	408	92	16.72	5.50	+	2.11E-40	2.87E-37
↳organic cyclic compound biosynthetic process	472	93	19.35	4.81	+	1.17E-36	2.28E-34
↳cellular nitrogen compound biosynthetic process	641	93	26.28	3.54	+	2.91E-27	1.36E-25
↳aromatic compound biosynthetic process	454	92	18.61	4.94	+	4.45E-37	1.01E-34
↳heterocycle biosynthetic process	458	93	18.77	4.95	+	1.33E-37	3.63E-35
↳gene expression	743	86	30.46	2.82	+	6.55E-19	1.98E-17
↳transcription by RNA polymerase II	201	63	8.24	7.65	+	1.05E-33	1.58E-31
transcription elongation from RNA polymerase II promoter	11	6	.45	13.31	+	3.01E-05	2.81E-04
↳DNA-templated transcription, elongation	15	6	.61	9.76	+	1.16E-04	1.01E-03
DNA replication checkpoint signaling	8	4	.33	12.20	+	9.05E-04	6.62E-03
↳DNA integrity checkpoint signaling	18	9	.74	12.20	+	4.96E-07	5.82E-06
↳cell cycle checkpoint signaling	30	11	1.23	8.94	+	3.13E-07	3.74E-06
↳intracellular signal transduction	124	16	5.08	3.15	+	1.10E-04	9.76E-04
↳signal transduction	158	18	6.48	2.78	+	1.76E-04	1.49E-03
↳cell communication	175	18	7.17	2.51	+	5.49E-04	4.25E-03
↳signaling	158	18	6.48	2.78	+	1.76E-04	1.50E-03
↳negative regulation of cell cycle phase transition	30	11	1.23	8.94	+	3.13E-07	3.77E-06
↳regulation of cell cycle phase transition	42	13	1.72	7.55	+	1.33E-07	1.64E-06
↳regulation of cell cycle process	47	14	1.93	7.27	+	6.43E-08	8.41E-07
↳regulation of cell cycle	70	14	2.87	4.88	+	4.03E-06	4.12E-05
↳negative regulation of cell cycle process	32	11	1.31	8.39	+	5.32E-07	6.13E-06
↳negative regulation of cell cycle	32	11	1.31	8.39	+	5.32E-07	6.19E-06
↳negative regulation of cellular process	103	20	4.22	4.74	+	5.00E-08	6.81E-07
↳negative regulation of biological process	133	20	5.45	3.67	+	1.90E-06	2.01E-05
negative regulation of transcription by RNA polymerase II	15	7	.61	11.38	+	1.39E-05	1.37E-04
↳negative regulation of transcription, DNA-templated	19	7	.78	8.99	+	4.67E-05	4.23E-04
↳negative regulation of nucleic acid-templated transcription	19	7	.78	8.99	+	4.67E-05	4.26E-04
↳negative regulation of RNA biosynthetic process	19	7	.78	8.99	+	4.67E-05	4.21E-04
↳negative regulation of macromolecule biosynthetic process	48	9	1.97	4.57	+	3.38E-04	2.79E-03
↳negative regulation of biosynthetic process	52	9	2.13	4.22	+	5.68E-04	4.34E-03
↳negative regulation of metabolic process	95	11	3.89	2.82	+	2.87E-03	1.90E-02
↳negative regulation of macromolecule metabolic process	89	10	3.65	2.74	+	5.34E-03	3.26E-02
↳negative regulation of RNA metabolic process	21	7	.86	8.13	+	7.85E-05	7.03E-04
↳negative regulation of nucleobase-containing compound metabolic process	23	8	.94	8.49	+	1.85E-05	1.81E-04
↳negative regulation of cellular metabolic process	62	11	2.54	4.33	+	1.15E-04	1.01E-03
↳negative regulation of nitrogen compound metabolic process	54	8	2.21	3.61	+	2.77E-03	1.85E-02
↳negative regulation of cellular biosynthetic process	52	9	2.13	4.22	+	5.68E-04	4.32E-03
↳negative regulation of cellular macromolecule biosynthetic process	48	9	1.97	4.57	+	3.38E-04	2.77E-03
DNA damage checkpoint signaling	13	5	.53	9.38	+	5.11E-04	3.97E-03
↳signal transduction in response to DNA damage	13	5	.53	9.38	+	5.11E-04	4.00E-03
tRNA wobble uridine modification	8	3	.33	9.15	+	7.87E-03	4.70E-02
DNA strand elongation involved in DNA replication	8	3	.33	9.15	+	7.87E-03	4.68E-02
DNA biosynthetic process	51	15	2.09	7.18	+	2.47E-08	3.54E-07
telomere maintenance	14	4	.57	6.97	+	4.64E-03	2.90E-02
↳telomere organization	14	4	.57	6.97	+	4.64E-03	2.88E-02
meiotic chromosome segregation	18	5	.74	6.78	+	1.71E-03	1.17E-02
↳nuclear chromosome segregation	50	9	2.05	4.39	+	4.41E-04	3.47E-03

↳chromosome segregation	52	9	2.13	4.22	+	5.68E-04	4.37E-03
↳meiotic cell cycle process	32	6	1.31	4.57	+	3.36E-03	2.14E-02
↳meiotic cell cycle	32	6	1.31	4.57	+	3.36E-03	2.12E-02
↳meiotic nuclear division	32	6	1.31	4.57	+	3.36E-03	2.13E-02
↳nuclear division	119	26	4.88	5.33	+	4.57E-11	8.19E-10
↳organelle fission	122	26	5.00	5.20	+	7.38E-11	1.30E-09
cell cycle phase transition	58	14	2.38	5.89	+	5.79E-07	6.62E-06
mitotic cell cycle checkpoint signaling	21	5	.86	5.81	+	3.03E-03	1.98E-02
↳negative regulation of mitotic cell cycle	21	5	.86	5.81	+	3.03E-03	1.99E-02
↳regulation of mitotic cell cycle	34	6	1.39	4.31	+	4.37E-03	2.75E-02
negative regulation of mitotic cell cycle phase transition	17	4	.70	5.74	+	8.27E-03	4.90E-02
mitotic sister chromatid segregation	35	7	1.43	4.88	+	1.12E-03	7.83E-03
↳mitotic nuclear division	101	23	4.14	5.56	+	3.18E-10	5.34E-09
↳sister chromatid segregation	39	8	1.60	5.00	+	4.28E-04	3.39E-03
microtubule cytoskeleton organization	26	5	1.07	4.69	+	6.67E-03	4.00E-02
Unclassified	3293	76	134.99	.56	-	1.46E-13	3.02E-12
small molecule metabolic process	395	4	16.19	.25	-	7.22E-04	5.40E-03
intracellular transport	292	2	11.97	.17	-	1.02E-03	7.32E-03
↳cellular localization	375	4	15.37	.26	-	9.77E-04	7.07E-03
↳localization	696	5	28.53	.18	-	7.64E-08	9.90E-07
↳transport	641	3	26.28	.11	-	1.14E-08	1.74E-07
↳establishment of localization	650	5	26.64	.19	-	3.59E-07	4.25E-06
protein transport	196	1	8.03	.12	-	4.85E-03	3.00E-02
↳nitrogen compound transport	285	2	11.68	.17	-	1.52E-03	1.05E-02
↳organic substance transport	330	2	13.53	.15	-	2.28E-04	1.93E-03
↳establishment of protein localization	199	1	8.16	.12	-	4.86E-03	2.99E-02
↳protein localization	239	1	9.80	.10	-	1.06E-03	7.55E-03
↳macromolecule localization	288	2	11.81	.17	-	1.01E-03	7.27E-03
transmembrane transport	202	1	8.28	.12	-	4.95E-03	3.03E-02
cellular protein localization	222	1	9.10	.11	-	2.32E-03	1.55E-02
↳cellular macromolecule localization	228	1	9.35	.11	-	1.55E-03	1.07E-02
carbohydrate derivative biosynthetic process	163	0	6.68	< 0.01	-	3.12E-03	2.03E-02
↳carbohydrate derivative metabolic process	208	1	8.53	.12	-	3.32E-03	2.14E-02
translational elongation	173	0	7.09	< 0.01	-	2.08E-03	1.41E-02
↳translation	173	0	7.09	< 0.01	-	2.08E-03	1.40E-02
↳peptide biosynthetic process	174	0	7.13	< 0.01	-	1.29E-03	9.01E-03
↳peptide metabolic process	187	0	7.67	< 0.01	-	8.61E-04	6.40E-03
↳cellular amide metabolic process	208	0	8.53	< 0.01	-	3.83E-04	3.12E-03
↳amide biosynthetic process	189	0	7.75	< 0.01	-	8.74E-04	6.43E-03
↳organonitrogen compound biosynthetic process	492	1	20.17	.05	-	4.35E-08	5.98E-07
vesicle-mediated transport	222	0	9.10	< 0.01	-	2.70E-04	2.24E-03
ribosome biogenesis	156	0	6.39	< 0.01	-	2.91E-03	1.92E-02
organophosphate biosynthetic process	145	0	5.94	< 0.01	-	4.37E-03	2.74E-02
↳organophosphate metabolic process	209	1	8.57	.12	-	3.33E-03	2.14E-02

About | Release Information | Contact Us | System Requirements | Privacy Policy | Disclaimer

© Copyright 2022 Paul Thomas All Rights Reserved.
